# Transcriptional Profiling of Tumorspheres Reveals TRPM4 as a Novel Stemness Regulator in Breast Cancer

**DOI:** 10.3390/biomedicines9101368

**Published:** 2021-10-01

**Authors:** John Verigos, Dimitris Kordias, Styliani Papadaki, Angeliki Magklara

**Affiliations:** 1 Institute of Molecular Biology and Biotechnology-Foundation for Research and Technology, 45110 Ioannina, Greece; ioan_ver@yahoo.gr (J.V.); d.kordias@hotmail.com (D.K.); 2 Department of Clinical Chemistry, Faculty of Medicine, University of Ioannina, 45110 Ioannina, Greece; st.papadaki7@yahoo.gr; 3 Institute of Biosciences, University Research Center of Ioannina (URCI), 45110 Ioannina, Greece

**Keywords:** breast cancer stem cells, mammospheres, tumorspheres, targeted therapy, combination therapy, stemness marker, RNA-sequencing, transient receptor potential cation channel subfamily M member 4 (*TRPM4*)

## Abstract

Cancer stem cells (CSCs) have been implicated in the development of chemoresistance, tumor recurrence and metastasis in breast cancer, thus emerging as a promising target for novel therapies. To identify novel stemness regulators that could potentially be targeted in luminal ER^+^ tumors, we performed RNA-sequencing (RNA-seq) in MCF-7 adherent monolayer cells and tumorspheres enriched in breast CSCs (bCSCs). We identified 1421 differentially expressed genes (DEGs), with 923 of them being upregulated and 498 downregulated in tumorspheres. Gene ontology and pathway enrichment analyses revealed that distinct gene networks underlie the biology of the two cell systems. We selected the transient receptor potential cation channel subfamily M member 4 (*TRPM4*) gene that had not been associated with cancer stemness before for further investigation. We confirmed that *TRPM4* was overexpressed in tumorspheres and showed that its knock-down affected the stemness properties of bCSCs in vitro. *TRPM4* inhibition revealed potential anti-tumor effects by directly targeting the bCSC subpopulation. We suggest that *TRPM4* plays a key role in stemness mediation, and its inhibition may represent a novel therapeutic modality against bCSCs contributing in the improvement of breast cancer treatments.

## 1. Introduction

Breast cancer is the most prevalent cancer worldwide, with 2.3 million women having been diagnosed with the disease and 685,000 patients having died of it in 2020 [1]. Conventional chemotherapy is still at the forefront of treatment regimens, being effective in inciting tumor remission, yet a considerable fraction of patients ends up developing resistance and suffering relapse overtime [2]. This occurs even in the least aggressive subtype of BC, the luminal ER^+^, which is treated with chemotherapy in advanced cases, and has a good prognosis in the initial 5 years after diagnosis, but has an increased chronic annual risk of recurrence thereafter [3]. More than half of the metastases of ER^+^ tumors occur 5 years or longer after diagnosis with some patients suffering recurrence after more than 20 years [4].

A possible explanation to account for this observation is based on the existence of rare tumor subpopulations with intrinsic resistance that survive treatment and drive tumor regrowth leading to patient relapse. Such tumor cells possess oftentimes stem-like characteristics, such as self-renewal and the capacity for differentiation, and are referred to as breast cancer stem cells (bCSCs). In breast, CSCs were first isolated from breast tumor specimens by Al-Hajj and colleagues [5]. The researchers determined that only a small fraction of cancer cells, those with the phenotype CD44^+^CD24^–/low^/lineage^–^, could initiate tumor formation, when transplanted to the mammary fat pad of severe combined immunodeficiency disease mice [5]. Later studies further confirmed that cells with this phenotype isolated from tumor samples or breast cancer cell lines possessed tumor-initiating properties and displayed increased resistance to conventional therapeutic schemes compared to the bulk of tumor cells [6,7]. As such, bCSCs have been implicated in the etiology of tumor recurrence and metastases [8] and their specific targeting has emerged as a promising approach complementary to the standard course of therapy, in order to improve clinical outcome in breast cancer [9].

To identify pharmacological targets in bCSCs, an in-depth characterization of the molecular mechanisms underpinning their biology is needed; however, the rarity of this tumor subpopulation has been a major hurdle for their study. Several methods of isolation and/or enrichment of bCSCs have been described in the literature [10]; the most widespread in vitro system for their study and manipulation is the generation of mammospheres, which are 3D-spheroid structures first developed by Dontu and colleagues for the propagation of human mammary epithelial cells under non-adherent, non-differentiated culture conditions [11]. These discrete cell clusters were enriched in progenitor cells capable of differentiating towards distinct mammary tissue lineages [11]. A later study showed that mammospheres cultured from breast cancer cell lines and primary tumors, also known as tumorspheres, were highly enriched in CD44^+^CD24^–/low^ cells [12]. Since then, numerous reports have meticulously characterized mammospheres/tumorspheres generated from various cancer cell lines and patient tumor samples confirming that each one is derived directly from the proliferative clonal expansion of a single tumor initiating cell and is enriched in cells that retain stem-like or progenitor cell properties [6,13,14]; thus, the 3D tumorsphere system represents a useful and reliable tool to study bCSCs in vitro.

In this study, we used next generation sequencing (NGS) to characterize the transcriptome of MCF-7-derived mammospheres and parental cells in an effort to identify novel regulators of stemness that could be potential targets in luminal ER^+^ tumors. Our bioinformatic analysis identified numerous differentially expressed genes including several known stemness markers validating our data, as well as novel genes that may play an important role in bCSCs. One of the latter ones was *TRPM4* a member of the TRPM family of ion channels that are overexpressed in various types of cancer [15]. *TRPM4* itself was recently shown to be upregulated in breast cancer [16], where it was associated with epithelial-to-mesenchymal transition (EMT) gene sets [16] spurring our interest to further investigate its role in bCSCs. Our results suggest that *TRPM4* is a novel bCSC-regulator, and its targeting may lead to a significant reduction of this aggressive subpopulation and may enhance therapeutic results in breast cancer.

## 2. Materials and Methods

### 2.1. Cell Lines and Pharmacological Agents

The MCF-7 human breast cancer cell line was purchased from ATCC (LCG standards, Middlesex, UK) and was cultured in Dulbecco’s modified Eagle’s medium (DMEM, high glucose) supplemented with 10% fetal bovine serum and 1% penicillin/streptomycin in a humidified atmosphere of 5% CO_2_ at 37 °C. Cells were routinely passaged every 2 or 3 days and tested for mycoplasma. The TRPM4 inhibitor used was 9-phenantrhol (Sigma-Aldrich St. Louis, MO, USA, cat. No. 211281). The chemotherapeutic drug used was doxorubicin (Adriblastina Hydrochloride 10 mg/5 mL VIAL Pfizer, New York, NY, USA).

### 2.2. TRPM4 Knock-Down

A small interfering RNA (siRNA) previously published [17] was used for transient *TRPM4* knock-down and was transfected into cells using Lipofectamine™ RNAiMAX Transfection Reagent (ThermoFisher Scientific, Waltham, MA, USA) according to manufacturer’s instructions. A scrambled siRNA was used as a negative control.

### 2.3. Mammosphere Formation Assay

MCF-7 cells at an early passage were resuspended in mammosphere medium (DMEM/F12 supplemented with B27 (Gibco, ThermoFisher Scientific, Waltham, MA, USA), EGF (20 ng/mL, Immunotools, Friesoythe; Germany), and FGF (20 ng/mL Immunotools, Friesoythe; Germany) and grown as described before [18]. Only spheres with a diameter over 50 µm were counted. The mammopshere formation efficiency (MFE) was calculated based on the following formula: (number of mammospheres per well/number of cells seeded per well) × 100 [13].

### 2.4. Flow Cytometry

Breast cancer cells were harvested (or dissociated from mammospheres) and were incubated with anti-CD44-PE (Cat. No. 550989) and anti-CD24-FITC (Cat. No. 560992) conjugated antibodies (both from BD Biosciences, San Jose, CA, USA) for 20 min at 4 °C in the dark. Cell staining was followed by two washes with PBS-2% FBS. Finally, the cells were centrifuged (1500 rpm, 5 min, 4 °C) and then resuspended in 200 µL PBS-2% FBS. Analysis was then performed using the BD FACS Aria II-BD Biosciences. Gates for fluorescence fractionations were established using unstained and isotype controls, PE-IgG (Cat. No: 21275514) and FITC-IgG (Cat. No. 21815013) both from Immunotools (Friesoythe; Germany).

### 2.5. Pharmacological Treatment

Dose–response experiments with the TRPM4 inhibitor 9-phenanthrol were performed with MCF-7 tumorspheres. The MFE was calculated at different time points (3–10 days) and FACS analysis was also performed to monitor the CD44^+^CD24^−/low^ bCSC-subpopulation. The most pronounced reduction in the MFE and in the number of bCSCs was achieved at 50 μΜ οf 9-phenanthrol after treatment for 7 days. For the combination treatment of tumorspheres with 9-phenanthrol and doxorubicin, preliminary experiments were performed to determine the administration scheme for the two reagents that yielded the maximum response at the lowest concentrations (data not shown). The final protocol applied involved pretreatment of tumorspheres with 50 µM of 9-phenanthrol for 5 days and addition of 2.5 µM doxorubicin for 2 more days.

### 2.6. Immunoblot Analysis

For Western blot analysis, total protein quantitation was performed using the BCA Protein Assay (ThermoScientific, Waltham, MA, USA). The antibodies used were anti-TRPM4 antibody (1:5000) (cat. No.TA500381, Origene, Rockville, MD, USA), anti-Actin (1:10,000) (a.a. 50–70, MAB1501, clone C4, Merck-Millipore Darmstadt, Germany), anti-rabbit IgG-HRP-linked (1:2000) (cat. No. 7074, Cell Signaling Technology, Danvers, MA, USA), and anti-mouse IgG-HRP-linked (1:2000) (cat. No. 7076, Cell Signaling Technology, Danvers, MA, USA).

### 2.7. Immunofluorescence Analysis

MCF-7 breast cancer cells were seeded in coverslips and the next morning they were washed with PBS and fixed with 4% PFA. The cells were incubated with the primary antibody against TRPM4 (1:50) (cat. No. TA500381, Origene, Rockville, MD, USA) for 1 h at room temperature (RT) and with the secondary antibody (Goat anti-Mouse IgG Alexa Fluor 488, 1:400) (cat. No. A-1100, ThermoFisher Scientific, Waltham, MA, USA)) for 45 min at RT. For mammosphere staining, first generation MCF-7 mammospheres were collected by centrifugation (800 rpm, 3 min, RT), washed in PBS (800 rpm, 3 min, RT) and fixed in 4% PFA for 30 min. The spheres were incubated with the primary antibody overnight at 4 °C and with the secondary antibody at RT for 45 min. In both cases, TOPRO-3 (cat. No. T3605, Invitrogen, ThermoFisher Scientific, Waltham, MA, USA) was used for DNA staining. A Leica SP5 confocal microscope was used to image the specimens. For comparison reasons, the same parameters (PMTs and offset) were employed for imaging, and the images obtained were processed in the same manner.

### 2.8. RNA Extraction, cDNA Synthesis and q-RT-PCR

For total RNA extraction the RNeasy Kit (Qiagen) was used. For total RNA extraction from mammospheres, first generation mammospheres were dissociated and replated in mammosphere forming conditions. Four days upon replating, spheres were collected by centrifugation (800 rpm, 3 min, RT) and processed for RNA extraction. Total RNA concentration and purity was measured with NanoDrop^TM^ 2000 (ThermoFisher Scientific, Waltham, MA, USA). For cDNA preparation the PrimeScript 1st strand cDNA Synthesis Kit (TAKARA, Kusatsu, Shiga, Japan) was used. For the q-RT-PCR experiments the KAPA SYBR^®^ FAST qPCR Kit Master Mix (2x) ( cat. No. KR0389 Sigma-Aldrich St. Louis, MO, USA was used.

### 2.9. RNA-Sequencing (RNA-seq) and Bioinformatic Analysis

RNA-seq libraries were prepared with the TruSeq RNA v2 kit (Illumina, San Diego, CA, USA) using 1 μg of total RNA. The libraries were checked with the Agilent bioanalyzer (DNA1000 chip) (Agilent, Santa Clara, CA, USA), quantitated with the qubit HS spectrophotometric method and pooled in equimolar amounts for sequencing. Approximately 25 million, 75 bp long, single-end reads were generated for each sample on an Illumina NextSeq500 or Novaseq 6000 sequencer. For each sample, two biologically independent replicates were sequenced yielding highly similar results.

Quality Control was performed with the fastq raw data file using the “FASTQC” software (GPL v.3, Babraham Institute, Cambridge, UK) FastQ files were aligned to the human genome (hg19) using HISAT2 (HISAN 3N-beta release) [19]. Counts were defined using the HTSeq htseq-count command with the “intersection non empty” option [20]. Normalization was performed with the estimate size factor function followed by Differentially Expressed Genes Analysis. The count files were used as input for DESeq2 (Bioconductor version: Release (3.13) [21] for identification of DEGs between mammospheres and adherent monolayer cells with a statistically significant cut-off value of *p* < 0.05. Additional cut-off criteria were set as fold-change ≥ 2, *p*-adjust < 0.01 and number of reads > 10.

All raw and processed data files have been submitted to GEO (accession number GSE182532).

The volcano plot was designed using GraphPad Prism 8.

### 2.10. Gene Ontology and Gene Set Enrichment Analysis of DEGs

Gene ontology (GO) analysis for the DEGs was performed using the PANTHER classification system (version 16) [22]. The annotation data set selected was PANTHER GO-Slim Biological Process. Only categories with a *p*-value < 0.05 (as determined by Fisher’s exact test) and an FDR < 0.05 were further analyzed. Biological processes that were underrepresented in our data, as well as the ones that had to do with neurogenesis, were excluded from further analysis.

Gene set enrichment analysis for the DEGs was also performed using the GSEA software [23,24]. The number of permutations were set to 1000, the permutation type was ‘gene_set’ and gene sets with <15 and >500 genes were excluded [25]. The gene set database selected was “hallmarks” that provides gene sets that are well-defined biological states or processes.

### 2.11. Statistical Analysis

Data presented were generated from at least three independent biological experiments, unless otherwise stated, and they are expressed as mean ± SE. An unpaired two-tailed Student’s *t*-test was used for statistical analysis.

## 3. Results

### 3.1. RNA Sequencing Data Analysis

Mammospheres or tumorspheres are 3D spheroids generated by breast cancer cells grown under non-adherent conditions and are significantly enriched in bCSCs [13,26]. We confirmed that this was also the case in our MCF-7 mammosphere system before proceeding with subsequent experiments. Indeed, these spheres exhibited an ~11-fold enrichment in the CD44^+^CD24^−/low^ bCSC-subpopulation (Supplementary Figure S1A,B) and overexpression of known stemness genes (Supplementary Figure S1C), when compared to MCF-7 cells grown in adherent monolayers.

**Figure 1:**
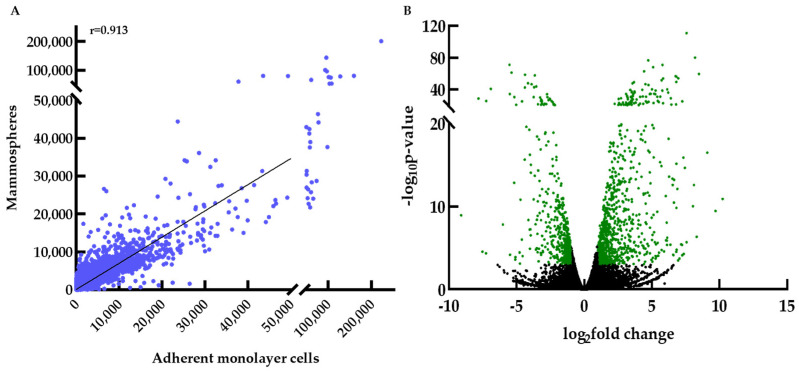
Gene expression in adherent monolayer cells and mammospheres. (**A**) Scatter plot of the 27,967 genes analyzed evaluated as average number of reads. The high Pearson correlation coefficient (r = 0.913) indicates similar expression profiles between the two cell systems, as expected. (**Β**) Volcano plot (*p*-value vs. log_2_fold change of gene expression) of the 27,967 genes that were analyzed. Differentially expressed genes (DEGs) in mammospheres with a ≥2-fold change are depicted in green (*p*-value < 0.05 and *p*-adjust value < 0.01).

We used this system to identify novel stemness regulators in breast cancer. For this purpose, we analyzed the transcriptome profiles of MCF-7 breast cancer cells grown as 2D monolayers or 3D spheres by RNA-seq. In total, we analyzed 27,967 genes that mostly exhibited a similar expression pattern between the two cell systems (Pearson correlation coefficient r = 0.913), as expected (Figure 1A). Mammospheres expressed 14,937 genes, with 496 of them being expressed only in this system, while adherent monolayer cells expressed 14,641 genes with 316 uniquely expressed genes (Supplementary Figure S2).

To assess the validity of our RNA-seq data, we identified the top 100 most highly expressed genes in the MCF-7 monolayer adherent cells and in the mammospheres (Supplementary Table S1) and cross-referenced some of them to published literature. In both cases the keratins *KRT8* and *KRT18,* which are considered typical luminal markers [27,28], were very highly expressed. We also detected elevated levels of *CSDE1*, which encodes for an RNA-binding protein that plays an important role in several cellular processes, such as cell cycle and apoptosis, and has been shown before to be overexpressed in MCF-7 cells [29]. High expression levels of the elongation factor 2 (*EEF2)* have been associated with poor prognosis in hormone receptor-positive breast cancer [30]. Other well-known breast cancer associated genes that were highly expressed in both cell systems included *GATA3* [31], *GNAS* [32], *FASN* [33] and *NCOA3* [34].

Technical validation of the RNA-seq data was performed by RT-qPCR experiments as described in the next section.

### 3.2. Identification and Validation of Differentially Expressed Genes

Next, we focused on identifying the DEGs between mammospheres and parental adherent cells, aiming to discover novel potential regulators of bCSCs. Their transcriptomes were compared using the R package DESEQ2 and DEGs were identified by setting a 2-fold-change and a *p*-value ≤ 0.05 (with *p*-adj ≤ 0.01) as cut-off values. In this manner, we identified 1421 DEGs with 923 of them being upregulated and 498 downregulated in mammospheres (Figure 1B). A complete list of these genes is provided in the Supplementary Table S2. Notably, among the most upregulated genes we find the well-known stemness marker *CXCR4* [35,36], *CYP1A1*, which was reported to control bCSCs proliferation, development and self-renewal [37], and *TM4SF1* that was shown to promote breast cancer stem cell traits [38]. In the downregulated genes, we find *CAV1*, which was recently reported to be downregulated in bCSCs and was associated with a metabolic switch from mitochondrial respiration to aerobic glycolysis [39] and *ELOVL2*, an enzyme involved in lipid metabolism with a reduced expression in a spheroid-induced EMT model [40].

**Figure 2:**
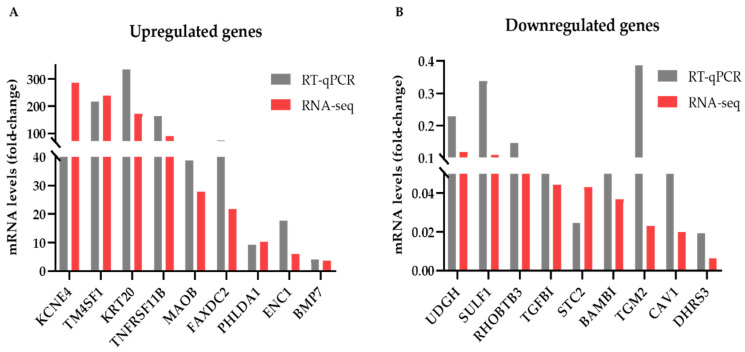
Differentially expressed genes and RT-qPCR validation. Comparison of the RNA-seq and RT-qPCR data for several (**A**) upregulated genes and (**B**) downregulated genes in MCF-7 mammospheres compared to monolayers. Representative data from 1 to 3 biological replicates with similar results are shown for the RT-qPCR experiments.

We also validated the results of our DEG analysis by assessing the expression levels of several DEGs by RT-qPCR. A side-by-side comparison of these results with the corresponding RNA-seq data is shown in Figure 2. We selected to examine genes that showed high (55–230), medium (22–28) or low (6–12) fold-change of expression levels in the mammospheres compared to monolayer cells in the RNA-seq data. Overall, the changes in mRNA levels detected by the two techniques were similar for most genes examined with the exception of *KCNE4* in the upregulated (Figure 2A) and of the *TGM2* in the downregulated genes (Figure 2B). In both cases, the RNA-seq experiments overestimated the fold-change of gene expression in the mammospheres compared to adherent cells; however, the pattern of expression (up or down) was verified.

### 3.3. Gene Ontology and Gene Set Enrichment Analysis for DEGs

To further investigate the biological significance of our data, we performed GO analysis for the DEGs, using the PANTHER classification system [22]. PANTHER GO-Slim biological process analysis was performed separately for the up- and down-regulated genes in mammospheres, using the statistical overexpression test.

We determined a total of 50 overrepresented biological processes in the upregulated genes and 33 in the downregulated genes in mammospheres, clustered in 8 and 5 major groups, respectively (Supplementary Table S3). Each group contained similar (or identical) biological processes, as recognized by the corresponding GO terms and GO terms grouped together included mostly common genes (data not shown). For simplicity and clarity reasons, we chose to illustrate one GO term per group, the one that contained the largest number of genes (Supplementary Figure S3). In the upregulated DEGs in MCF-7 mammospheres, the most numerous groups were “signaling” and “developmental process” (Supplementary Figure S3A)**,** while in the downregulated DEGs we had “cellular component organization or biogenesis” and “developmental process” (Supplementary Figure S3B).

**Figure 3:**
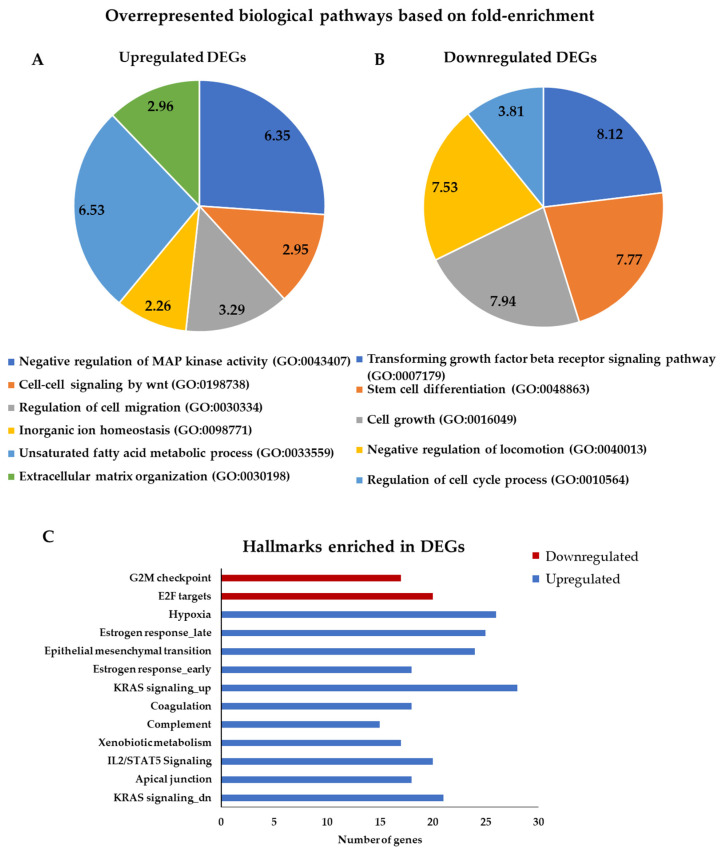
Gene ontology and hallmark analysis for DEGs in MCF-7 mammospheres. The top overrepresented biological pathways for the (**A**) upregulated and (**B**) downregulated DEGs resulting from PANTHER analysis. The fold-enrichment is shown in the diagrams. (**C**) The enriched hallmarks for the up- and downregulated DEGs resulting from the GSEA analysis.

For a more informative and biologically relevant depiction of our data, we also ranked the biological processes by fold-enrichment with a cut-off value of 2. Figure 3 illustrates the GO term/biological pathway that had the highest fold-enrichment score in each group. The most overrepresented biological pathway in the upregulated genes was the “unsaturated fatty acid metabolic process” (Figure 3A). The altered metabolism is one of the hallmarks of cancer. It has been reported that CSCs have increased needs in fatty acids and that unsaturated fatty acids maintain cancer cell stemness [41]. The Wnt pathway, an established CSC-promoting signaling cascade [42], was also highly enriched in the upregulated DEGs (Figure 3A). “Regulation of cell migration” was another overexpressed pathway in our analysis, a property that in CSCs has been correlated with metastasis [43]. Migration of cells is affected by the extracellular matrix (ECM) distribution and it has been reported that ECM of CSCs differs in composition compared to normal cells, playing a role in cancer stemness [44]. Divalent metal ions have a major role in tumor progression through modulation of the structure and function of various components of the ECM [45]. Consequently, the associated GO terms/biological pathways that we found enriched in the upregulated DEGs (Figure 3A) are in accordance with previous studies, further validating our results.

In the downregulated DEGs, the most overrepresented biological pathway was the “transforming growth factor beta receptor signaling pathway” (Figure 3B). Transforming have shown that TGFβ suppresses breast cancer tumorigenesis by reducing the CSC pools or by promoting the differentiation, whereas other studies have reported to promote or sustain stemness of the pool of CSCs in breast cancer [46]. In addition, “stem cell differentiation”, “cell growth”, “regulation of cell cycle process” and “negative regulation of locomotion” pathways were overrepresented in the downregulated DEGs, as one would expect to find in a cell population enriched for slowly proliferating, metastasis-prone CSCs (Figure 3B).

Next, we performed hallmark analysis, using the GSEA software [23,24], implementing separate analysis for the up- and downregulated genes in mammospheres. The analysis revealed 11 enriched gene sets in the upregulated DEGs and 2 enriched gene sets in the downregulated DEGs in mammospheres (Figure 3C and Supplementary Table S4). It is noteworthy that EMT and hypoxia, two of the most widely recognized CSC hallmarks, were among the most represented ones in the upregulated DEGs.

Taken together the results described above support the validity of our RNA-seq data and particularly the use of mammospheres as a discovery tool for novel breast cancer stemness regulators.

### 3.4. TRPM4 Is Overexpressed in MCF-7 Mammospheres

The GO analysis of the upregulated genes in the MCF-7 mammospheres confirmed overrepresentation of several well-known stemness associated pathways, but also identified one that, to our knowledge, has not been extensively studied in bCSCs before. This was “inorganic ion homeostasis” (Figure 3A), which was clustered with similar biological processes under the more general GO term “ion transport” (Supplementary Table S3). Manual inspection of the genes in this group revealed several genes that had not been associated with cancer stemness before, one of them being the *TRPM4* gene, a member of the TRPM family of ion channels [15]. *TRPM4* is known to be overexpressed in breast [16] and other types of cancer [47]. We confirmed these data by interrogating the Oncomine data base [48]. We found that *TRPM4* was overexpressed in aggressive breast tumors in two separate patient data sets (Supplementary Figure S4). These findings prompted us to investigate further its role in bCSCs.

**Figure 4:**
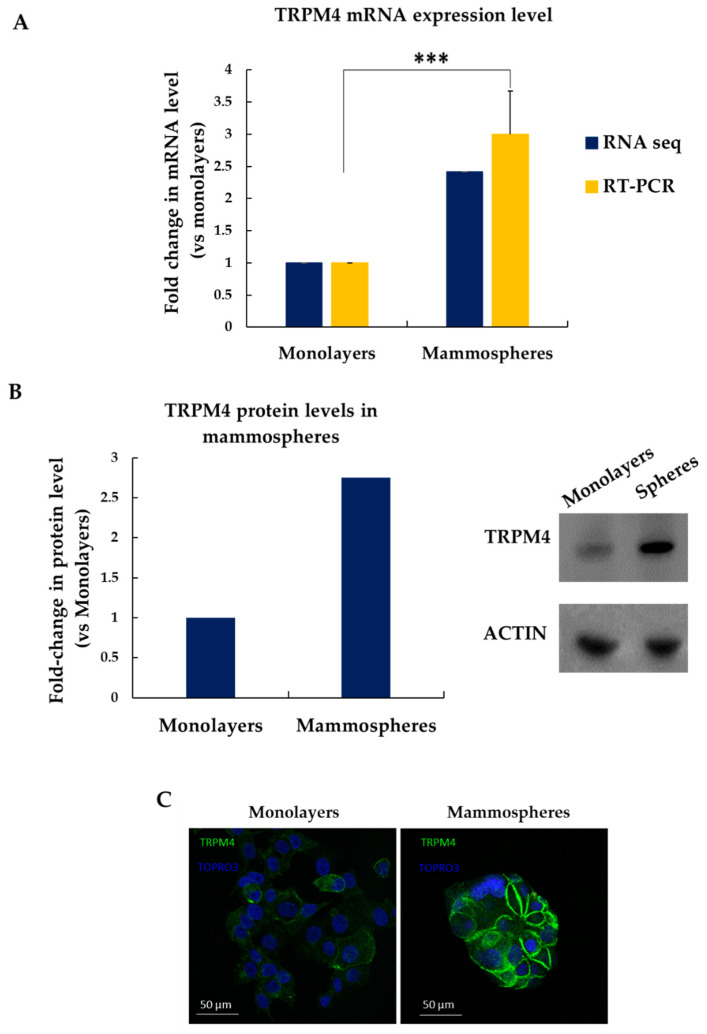
TRPM4 is overexpressed in MCF-7 mammospheres. (**A**) TRPM4 mRNA levels in adherent cells grown in monolayers or mammospheres as estimated by RNA-seq and RT-qPCR. (**B**) Western blot of protein lysates from MCF-7 monolayers and mammospheres with an antibody against TRPM4 or actin (control) (left). Quantification of Western blot using Image J. (**C**) Immunofluorescence of MCF-7 monolayers and mammospheres stained with an antibody against TRPM4 protein (green) and TORPO3 (blue). Error bars represent the SEM of biological replicates (*n* = 2–4). ***: *p* < 0.001. Scale bars represent 50 μm.

Firstly, we sought to confirm our RNA-seq data, which showed that TRPM4 mRNA expression levels were 2.4-fold higher in MCF-7 mammospheres compared to the adherent cells grown in monolayers (Figure 4A, blue bars). Indeed, RT-qPCR experiments validated these findings, showing a ~3-fold overexpression of TRPM4 in mammospheres (Figure 4A, yellow bars). Western blot experiments showed that TRPM4 protein levels were ~2.7-fold higher in the MCF-7-derived mammospheres compared to the corresponding attached cells (Figure 4B). Immunostaining of cells grown in monolayers or mammospheres with an antibody against TRPM4 further corroborated its overexpression in the latter ones and showed that it is mostly localized on the cell membrane as expected (Figure 4C).

Overall, these data demonstrate convincingly that TRPM4 is overexpressed in MCF-7 mammospheres both on the mRNA and protein level.

### 3.5. TRPM4 Regulates the Stemness Properties of bCSCs In Vitro

The upregulation of *TRPM4* expression in the MCF-7 mammospheres, known to be highly enriched in bCSCs, urged us to investigate its potential role in the regulation of this subpopulation.

**Figure 5:**
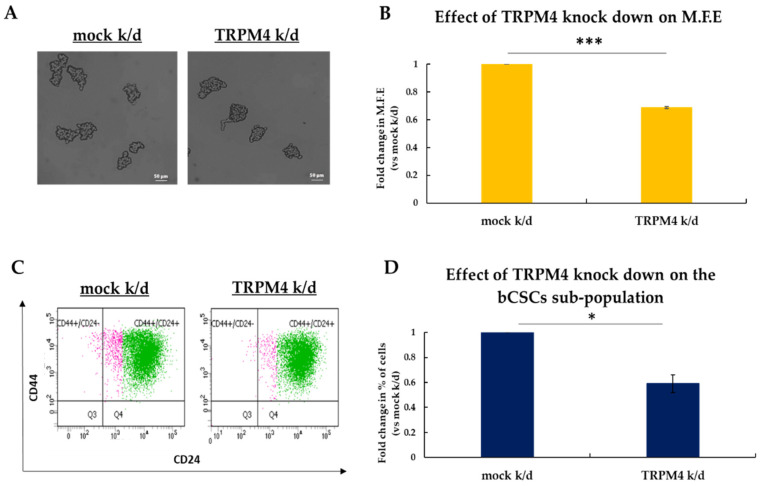
Effects of TRPM4 knock down on the bCSCs sub-population. (**A**) Representative images of MCF-7 mammospheres upon TRPM4 siRNA-mediated knock-down. Cells transfected with scrambled siRNA served as the control. (**B**) Effect of TRPM4 knock-down on the mammosphere forming efficiency (MFE). MCF-7 cells were transfected with siRNA against *TRPM4* and cultured under mammosphere-forming conditions for 7 days when the MFE was calculated. (**C**) Graphical representation of the FACS analysis for CD44^+^CD24^−/low^ cells in MCF-7 mammospheres after siRNA-mediated TRPM4 knock-down. (**D**) Quantification of FACS analysis presented in (**C**). Error bars represent SEM. * *p* < 0.05, *** *p* < 0.001. Scale bars represent 50 μm.

To this end, we knocked down *TRPM4* expression in MCF-7 cells (Supplementary Figure S5) using a previously published siRNA [17] and examined its effects on the stemness properties of bCSCs by applying the mammosphere-forming assay (Figure 5A,B). The results of these experiments revealed that TRPM4 knock-down resulted in the formation of a significantly smaller number of mammospheres compared to control (Figure 5A). Specifically, the TRPM4 siRNA-treated cells had 32% lower efficiency in forming mammospheres compared to the scramble siRNA-treated ones (Figure 5B). To further show that the decreased mammosphere forming efficiency was a specific effect of the TRPM4 knock-down on the bCSCs, we performed FACS analysis to monitor the CD44^+^CD24^−/low^ CSC subpopulation. The last day of the experiment the mammospheres in each condition were collected, dissociated and stained against the surface markers CD44 and CD24 (Figure 5C). FACS analysis showed 40% reduction in the percentage of the CD44^+^CD24^−/low^ CSC subpopulation upon TRPM4 knock-down compared to control cells (Figure 5D).

The above data strongly support the role of TRPM4 as a key regulator of the stemness properties of bCSCs in vitro.

Stemness-associated signaling cascades, such as the Wnt, Hedgehog and Notch pathways, regulate the maintenance of bCSCs (reviewed in [49]). Some of them are also involved in the induction of EMT that has been associated with the generation of bCSCs (reviewed in [50]). To gain some insight into the mechanisms that TRPM4 employs to regulate breast cancer stemness, we selected several molecules that participate in these pathways and showed considerable expression in our system and interrogated their mRNA levels in *TRPM4* k/d MCF-7 tumorspheres by RT-qPCR. The results are presented in Supplementary Figure S6. Among the EMT-associated molecules examined (blue in Figure S6), we found that the knock-down of TRPM4 led to significantly reduced mRNA levels of vimentin, an EMT marker. However, we did not see any changes in the levels of *CDH1*, which encodes for e-cadherin, or in the transcription factors *SNAI1* and *TWIST*. Among the Wnt-associated molecules examined (green in Figure S6), *PRICKLE1* was significantly downregulated in the *TRPM4* k/d MCF-7 tumorspheres. This gene is a member of the non-canonical Wnt/planar cell polarity (PCP) pathway and was recently described to be involved in breast cancer cell dissemination [51]. We did not observe any changes in the expression levels of *HHIP* (yellow in Figure S6) or *NOTCH1* (orange in Figure S6) involved in the Hedgehog and Notch signaling pathways, respectively.

### 3.6. TRPM4 Is a Potential Druggable Target in bCSCs

As mentioned above, several studies have reported on the overexpression of *TRPM4* in different types of cancer [52,53,54,55], and even though, its precise mechanism of action in tumors is currently unknown, it is already portrayed as a promising anticancer drug target [47].

Bearing this in mind and based on our data described above, we hypothesized that pharmacological inhibition of TRPM4 could, potentially, have anti-tumor effects through targeting the bCSC-subpopulation.

**Figure 6:**
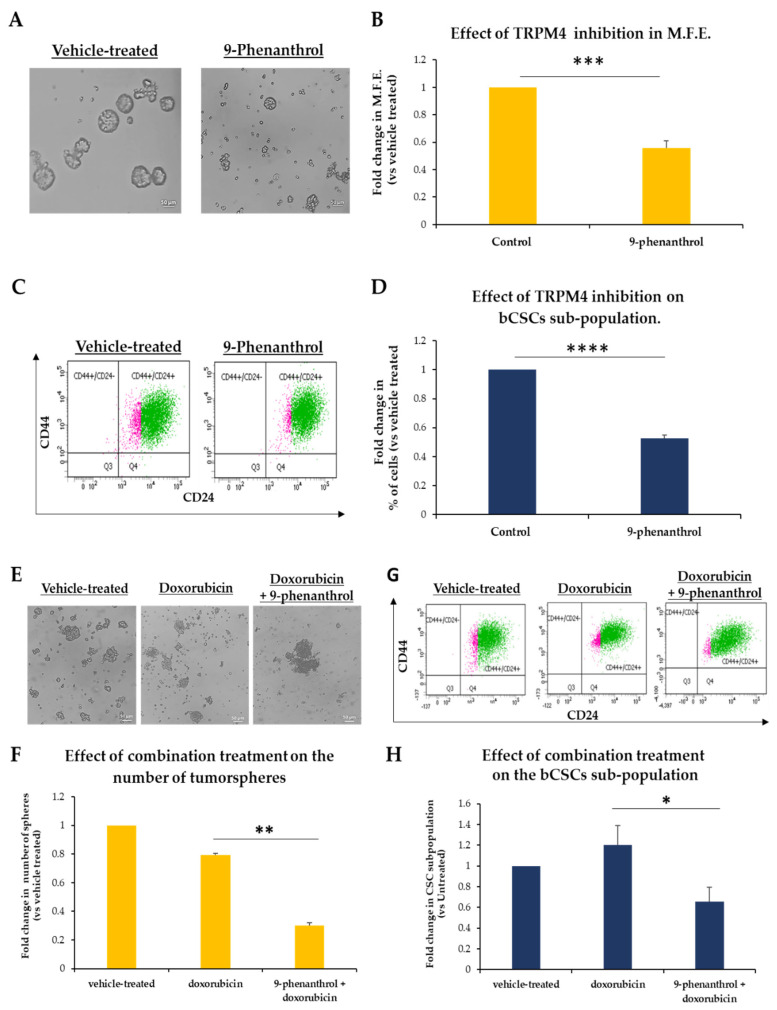
Targeting TRPM4 in bCSCs. (**A**) Representative images of MCF-7 mammospheres after inhibition of TRPM4 with 9-Phenantrhol (50 μΜ) for 7 days. Vehicle-treated spheres served as the control. (**B**) Graphical representation of the effects of TRPM4 inhibition on the MFE of MCF-7 mammosphere-derived single cells. The cells were treated with 9-Phenantrhol (50 μΜ) for 7 days, and on the last day, the number of formed mammospheres was counted and the MFE was calculated. (**C**) FACS analysis for the CD44 and CD24 cell surface markers was performed for the mammosphere-derived single cells after inhibitor treatment. Vehicle-treated cells were used as control. (**D**) Quantification of FACS analysis data presented in (**C**). (**E**) MCF-7 tumorspheres treated for 5 days with 9-Phenanthrol (50 μM). On the sixth day, doxorubicin (2.5 μΜ) was added for two more days. after treatment with doxorubicin (2.5 μΜ) or combination treatment on the number of MCF-7 tumorspheres. Representative images of tumorspheres on the last day of the treatment. (**F**) Graphical representation of the combination treatment on the number of tumorspheres. (**G**) FACS analysis for the CD44 and CD24 cell surface markers was performed for the tumorpshere-derived single cells after treatment. (**H**) Graphical representation of FACS analysis data for the CD44^+^CD24^−/low^ CSC subpopulation in MCF-7 tumorspheres. Error bars represent SEM. *: *p* < 0.05, **: *p* < 0.01, ***: *p* < 0.001, ****: *p* < 0.0001. Scale bars represent 50 μm.

To this end, we examined whether 9-phenanthrol, a TRPM4 channel blocker, could affect bCSCs. MCF-7 mammospheres were dissociated to single cells, re-plated under mammosphere-forming conditions and treated with 50 μΜ of the inhibitor for 7 days; at the end of treatment, the MFE was calculated (Figure 6A,B). Inhibition of TRPM4 resulted in 45% decrease in the number of mammospheres compared to vehicle-treated one (Figure 6A,B). At the same time, FACS analysis of the inhibitor-treated mammospheres showed an approximately 50% decrease in the CD44^+^CD24^−/low^ CSC-subpopulation compared to control mammospheres (Figure 6C,D).

These results strongly suggest that TRPM4 inhibition diminishes the stemness potential of bCSCs and targets directly this subpopulation in MCF-7 mammospheres. Notably, TRPM4 pharmacological inhibition phenocopied the effects of the siRNA-mediated knock-down, as described previously (Figure 5). Therefore, blocking of this ion channel may serve as a novel therapeutic modality for targeting the associated stemness pathways in bCSCs.

As it has been suggested before [56] and we have also shown [18], both a CSC-targeted agent and a conventional cytotoxic drug are needed to achieve significant tumor reduction and, presumably, improve cancer treatment. Therefore, we sought to investigate whether TRPM4 inhibition in combination with doxorubicin treatment could have such an effect. MCF-7-derived tumorspheres were treated for 5 days with 9-phenanthrol (50 μΜ) and for an additional two days with doxorubicin (2.5 μM). On the last day of treatment, the number of tumorspheres was counted (Figure 6E,F). Monotreatment with the chemotherapeutic agent led only to a 20% reduction in the number of spheres; on the other hand, pre-treatment with the TRPM4 inhibitor followed by doxorubicin administration led to a 70% decrease in the number of spheres compared to the vehicle-treated ones (Figure 6E,F). These results show that combination of a chemotherapeutic drug and a TRPM4 inhibitor is more successful in eliminating breast tumorspheres, probably because both stem/progenitor and differentiated cancer cells are targeted effectively. This was further confirmed by FACS experiments that were carried out in tumorspheres during the last day of treatment in order to monitor the CD44^+^CD24^−/low^ CSC-subpopulation (Figure 6G,H). Doxorubicin treatment alone could not target the CSC-pool and it rather enriched the culture in bCSCs. However, its combination with the TRPM4 inhibitor was efficient in reducing the size of the CD44^+^CD24^−/low^ CSC subpopulation by 35% (Figure 6G,H).

In conclusion, our data suggest that *TRPM4* is a mediator of stemness in breast cancer in vitro and its inhibition may hold promise as an efficient complementary therapeutic approach along with standard treatments.

## 4. Discussion

Current anti-cancer therapeutic schemes have been mostly designed based on the prevalent tumor lesions identified through the employment of genome-wide sequencing techniques that analyze the bulk of the tumor. This strategy, while useful, ignores the molecular aberrations of small but, potentially, aggressive tumor subpopulations that may have different drug sensitivities [57]. Such subpopulations are often endowed with stemness properties and are referred to as cancer stem cells. More specifically in breast cancer, CSCs have been described as manifesting remarkable resistance to standard treatments [58,59], making them prime candidates for the development of novel, targeted therapies that could be administered in combination with commonly used drugs, aiming to achieve complete tumor elimination [60,61].

To develop such therapies, the first crucial step is to isolate bCSCs for further characterization and manipulation in vitro. Among the methods that have been developed to enrich for bCSCs, the mammosphere or tumorsphere culture system has been established as a particularly advantageous model for their investigation [10]. In this study, we used the widely applied and extensively characterized 3D mammosphere culture system developed from the luminal ER^+^ breast cancer MCF-7 cells. Notably, this is one of the few cell lines where the CD44^+^C24^−/low^ bCSC-subpopulation has been isolated, transplanted into mice and shown to possess stemness properties [62]. We used this system to employ a non-biased genome-wide approach for the identification of the transcriptional networks that are distinct between cells grown in 2D monolayers and 3D spheres and are potentially essential for the maintenance of the CSC-pool in tumors. The quality of our sequencing data was extensively validated by RT-qPCR experiments; both methodologies yielded similar results regarding the fold-change in mRNA levels between monolayers and mammospheres for most genes examined. We also performed a literature search for some of the genes that were found to be overexpressed in both cell systems and we confirmed that they were breast cancer-associated ones according to several published reports [27,28,29,30,31,32,33,34]. We also performed DEG analysis. Among the most highly upregulated genes in mammospheres we detected several known stemness-associated genes [35,36,37,38]. Other highly upregulated genes in mammospheres included the ion channel *KCNE4* and the monoamine oxidase *MOAB*, which have not been associated with cancer stemness before. Interestingly, both genes have been found to be overexpressed in glioblastoma [63,64] a tumor type highly enriched in CSCs [65].

Our gene ontology analysis offered further validation of our data, but also provided us with new insights into the biology of bCSCs and enriched our record of potential specific targets. A stark example was the “unsaturated fatty acid metabolism”, which emerged as the most overrepresented GO term in the upregulated genes in mammospheres. This is in accordance with recent literature that shows that CSCs require more unsaturated fatty acids than their non-stem counterparts (reviewed in [66]) and thus, they overexpress the related metabolic pathways. However, not all key genes involved have been identified and studied yet. Several such candidate genes are listed in our supplementary data.

Probably the least studied pathways in CSCs are the ones regarding ion transport and ion homeostasis, even though recent studies have demonstrated that ion channels are present in various CSCs (reviewed in [67]). Thus, we turned our interest to these gene lists in an effort to discover novel CSC-regulators. We hypothesized that by identifying and modulating such genes, we might be able to suppress the bCSC-population and highlight a new therapeutic avenue in BC. We identified *TRPM4* as an overexpressed ion channel that had not been associated with cancer stem cells before. This gene is one of the 8 members of the TRPM (transient receptor potential melastatin) family, which belongs to the superfamily of TRP cation channels [15]. The TRPM family members are widely expressed transmembrane proteins that contribute to intracellular calcium homeostasis by promoting Ca^2+^ entry into the cytosol in response to various stimuli with the exception of *TRPM4* and 5 that are Ca^2 +^ impermeable [15]. The TRPM4 channel is only permeable to monovalent cations, mostly to Na^+^ and K^+^, upon activation by an increase in intracellular Ca^2 +^ [15]. Most interestingly to us, TRPM4 protein levels are increased in a variety of tumor types compared to healthy tissue, where it contributes to important cancer features, such as increased proliferation and migration and cell cycle shift (reviewed in [15,47]). Particularly in breast cancer, two recent studies showed that both the mRNA and protein levels of *TRPM4* were upregulated in specimens from breast cancer patients [16,55]. Increased TRPM4 staining intensity in the membrane and cytosol of tumor cells was significantly correlated with a worse patient prognosis [55]. These data were also confirmed by our own Oncomine analysis that showed that *TRPM4* overexpression is associated with aggressive breast tumors in the public datasets examined.

Taking into account these reports as well as the pathway analysis of our RNA-seq data, we hypothesized that *TRPM4* might play an important role in the regulation of breast CSCs. After confirming the overexpression of *TRPM4* in MCF-7 mammospheres, we set out to examine its functional effects on the same model of CSC-enrichment by knocking-down its gene expression. *TRPM4* silencing reduced the CSC fraction and impaired its stemness properties, suggesting that this ion channel is critical for CSC maintenance and activity in breast cancer. These results were also reproduced, when an TRPM4 inhibitor, 9-phenanthrol, was used. Our experiments with the combined use of 9-phenanthrol and doxorubicin in an in vitro tumorsphere system showed that this administration scheme was more efficient in reducing the number of spheres than the drug alone, which could not target the bCSC-subpopulation. This is in agreement with our previous results [18] and the view expressed by others [47] that both a CSC-specific agent and a chemotherapeutic drug are needed for complete tumor elimination.

It should be mentioned that 9-phenanthrol also inhibits TMEM16A/ANO1 [68], a calcium-activated chloride channel that is expressed in very low levels in our cells according to our RNA-seq data (data not shown) and probably not on the protein level. Hence, it is safe to assume that the effects observed in our cell system after 9-phenanthrol treatment are due solely to inhibition of the TRPM4 channel.

The precise mechanisms of action of *TRPM4* in cancer are currently under investigation [47]. Our results suggest that it may function through maintaining the CSC-compartment in tumors. The pathways that mediate TRPM4 activity in cancer stemness are yet unknown; our preliminary data suggest that this ion-channel may be involved in the EMT process. In support of this notion, recent studies have highlighted the connection of *TRPM4* expression with activation of the EMT in prostate [54] and breast [16] cancer cells, a process that has long been associated to cancer stemness [69]. *TRPM4* may also be involved in the regulation of the Wnt/PCP signaling pathway, which is often aberrantly expressed in cancer [70]. However, these findings need further investigation. As we could examine only a small set of stemness-associated genes by RT-qPCR, it is plausible that other signaling cascades also mediate *TRPM4* actions.

Our results also suggest that *TRPM4* can be used as a druggable target in breast cancer. Indeed, the cell membrane localization of the protein renders it an ideal candidate for novel anti-cancer drugs that could either fall into the small-molecule inhibitors category [71] or they could be specific antibodies that block the protein [72].

Future studies including in vivo experiments will clarify the role of *TRPM4* in cancer, will elucidate the regulatory mechanisms it employs to sustain CSCs and will evaluate its potential as a therapeutic target in breast tumors.

## Data Availability

All raw and processed data files have been submitted to GEO (accession number GSE182532).

## Supplementary Materials

The following are available online at https://www.mdpi.com/article/10.3390/biomedicines9101368/s1: Figure S1. MCF-7 mammospheres are highly enriched in bCSCs, Figure S2. Expressed genes in MCF-7 cells grown as adherent monolayers or mammospheres, Figure S3. Gene ontology analysis for DEGs, Figure S4. Oncomine analyses of breast cancer databases, Figure S5. Western blot analysis for TRPM4 siRNA knock-down in MCF-7 cells, Figure S6. mRNA expression levels of genes associated with stemness pathways in *TRPM4* k/d MCF-7 mammospheres. Table S1. The top 200 genes expressed in MCF-7 adherent monolayer cells and mammospheres. Table S2. The 1421 differentially expressed genes (DEGs) between adherent monolayer cells and mammospheres identified by RNA-sequencing, Table S3. The genes of each biological pathway overrepresented in DEGs, Table S4. The genes of the enriched hallmarks in DEGs.

## References

[1] World Health Organization (WHO) https://www.who.int/news-room/fact-sheets/detail/breast-cancer.

[2] Ji X., Lu Y., Tian H., Meng X., Wei M., Cho W.C. (2019). Chemoresistance mechanisms of breast cancer and their countermeasures. Biomed. Pharm..

[3] Ignatov A., Eggemann H., Burger E., Ignatov T. (2018). Patterns of breast cancer relapse in accordance to biological subtype. J. Cancer Res. Clin. Oncol..

[4] Pan H., Gray R., Braybrooke J., Davies C., Taylor C., McGale P., Peto R., Pritchard K.I., Bergh J., Dowsett M. (2017). 20-Year Risks of Breast-Cancer Recurrence after Stopping Endocrine Therapy at 5 Years. N. Engl. J. Med..

[5] Al-Hajj M., Wicha M.S., Benito-Hernandez A., Morrison S.J., Clarke M.F. (2003). Prospective identification of tumorigenic breast cancer cells. Proc. Natl. Acad. Sci. USA.

[6] Fillmore C.M., Kuperwasser C. (2008). Human breast cancer cell lines contain stem-like cells that self-renew, give rise to phenotypically diverse progeny and survive chemotherapy. Breast Cancer Res. BCR.

[7] Ablett M.P., Singh J.K., Clarke R.B. (2012). Stem cells in breast tumours: Are they ready for the clinic?. Eur. J. Cancer.

[8] Geng S.Q., Alexandrou A.T., Li J.J. (2014). Breast cancer stem cells: Multiple capacities in tumor metastasis. Cancer Lett..

[9] Saygin C., Matei D., Majeti R., Reizes O., Lathia J.D. (2019). Targeting Cancer Stemness in the Clinic: From Hype to Hope. Cell Stem Cell.

[10] Akbarzadeh M., Maroufi N.F., Tazehkand A.P., Akbarzadeh M., Bastani S., Safdari R., Farzane A., Fattahi A., Nejabati H.R., Nouri M. (2019). Current approaches in identification and isolation of cancer stem cells. J. Cell Physiol..

[11] Dontu G., Abdallah W.M., Foley J.M., Jackson K.W., Clarke M.F., Kawamura M.J., Wicha M.S. (2003). In vitro propagation and transcriptional profiling of human mammary stem/progenitor cells. Genes Dev..

[12] Ponti D., Costa A., Zaffaroni N., Pratesi G., Petrangolini G., Coradini D., Pilotti S., Pierotti M.A., Daidone M.G. (2005). Isolation and in vitro propagation of tumorigenic breast cancer cells with stem/progenitor cell properties. Cancer Res..

[13] Shaw F.L., Harrison H., Spence K., Ablett M.P., Simoes B.M., Farnie G., Clarke R.B. (2012). A detailed mammosphere assay protocol for the quantification of breast stem cell activity. J. Mammary Gland. Biol. Neoplasia.

[14] Wang R., Lv Q., Meng W., Tan Q., Zhang S., Mo X., Yang X. (2014). Comparison of mammosphere formation from breast cancer cell lines and primary breast tumors. J. Thorac. Dis..

[15] Hantute-Ghesquier A., Haustrate A., Prevarskaya N., Lehen’kyi V. (2018). TRPM Family Channels in Cancer. Pharmaceuticals.

[16] Wong K.K., Hussain F.A. (2020). TRPM4 is overexpressed in breast cancer associated with estrogen response and epithelial-mesenchymal transition gene sets. PLoS ONE.

[17] Holzmann C., Kappel S., Kilch T., Jochum M.M., Urban S.K., Jung V., Stockle M., Rother K., Greiner M., Peinelt C. (2015). Transient receptor potential melastatin 4 channel contributes to migration of androgen-insensitive prostate cancer cells. Oncotarget.

[18] Verigos J., Karakaidos P., Kordias D., Papoudou-Bai A., Evangelou Z., Harissis H.V., Klinakis A., Magklara A. (2019). The Histone Demethylase LSD1/KappaDM1A Mediates Chemoresistance in Breast Cancer via Regulation of a Stem Cell Program. Cancers.

[19] Kim D., Langmead B., Salzberg S.L. (2015). HISAT: A fast spliced aligner with low memory requirements. Nat. Methods.

[20] Anders S., Pyl P.T., Huber W. (2015). HTSeq--a Python framework to work with high-throughput sequencing data. Bioinformatics.

[21] Love M.I., Huber W., Anders S. (2014). Moderated estimation of fold change and dispersion for RNA-seq data with DESeq2. Genome Biol..

[22] Mi H., Muruganujan A., Thomas P.D. (2013). PANTHER in 2013: Modeling the evolution of gene function, and other gene attributes, in the context of phylogenetic trees. Nucleic Acids Res..

[23] Mootha V.K., Lindgren C.M., Eriksson K.F., Subramanian A., Sihag S., Lehar J., Puigserver P., Carlsson E., Ridderstrale M., Laurila E. (2003). PGC-1alpha-responsive genes involved in oxidative phosphorylation are coordinately downregulated in human diabetes. Nat. Genet..

[24] Subramanian A., Tamayo P., Mootha V.K., Mukherjee S., Ebert B.L., Gillette M.A., Paulovich A., Pomeroy S.L., Golub T.R., Lander E.S. (2005). Gene set enrichment analysis: A knowledge-based approach for interpreting genome-wide expression profiles. Proc. Natl. Acad. Sci. USA.

[25] Reimand J., Isserlin R., Voisin V., Kucera M., Tannus-Lopes C., Rostamianfar A., Wadi L., Meyer M., Wong J., Xu C. (2019). Pathway enrichment analysis and visualization of omics data using g:Profiler, GSEA, Cytoscape and EnrichmentMap. Nat. Protoc..

[26] Grimshaw M.J., Cooper L., Papazisis K., Coleman J.A., Bohnenkamp H.R., Chiapero-Stanke L., Taylor-Papadimitriou J., Burchell J.M. (2008). Mammosphere culture of metastatic breast cancer cells enriches for tumorigenic breast cancer cells. Breast Cancer Res. BCR.

[27] Abd El-Rehim D.M., Pinder S.E., Paish C.E., Bell J., Blamey R.W., Robertson J.F., Nicholson R.I., Ellis I.O. (2004). Expression of luminal and basal cytokeratins in human breast carcinoma. J. Pathol..

[28] Dai X., Cheng H., Bai Z., Li J. (2017). Breast Cancer Cell Line Classification and Its Relevance with Breast Tumor Subtyping. J. Cancer.

[29] Fang H., Yue X., Li X., Taylor J.S. (2005). Identification and characterization of high affinity antisense PNAs for the human unr (upstream of N-ras) mRNA which is uniquely overexpressed in MCF-7 breast cancer cells. Nucleic Acids Res..

[30] Meric-Bernstam F., Chen H., Akcakanat A., Do K.A., Lluch A., Hennessy B.T., Hortobagyi G.N., Mills G.B., Gonzalez-Angulo A. (2012). Aberrations in translational regulation are associated with poor prognosis in hormone receptor-positive breast cancer. Breast Cancer Res. BCR.

[31] Mehra R., Varambally S., Ding L., Shen R., Sabel M.S., Ghosh D., Chinnaiyan A.M., Kleer C.G. (2005). Identification of GATA3 as a breast cancer prognostic marker by global gene expression meta-analysis. Cancer Res..

[32] Garcia-Murillas I., Sharpe R., Pearson A., Campbell J., Natrajan R., Ashworth A., Turner N.C. (2014). An siRNA screen identifies the GNAS locus as a driver in 20q amplified breast cancer. Oncogene.

[33] Menendez J.A., Lupu R. (2017). Fatty acid synthase regulates estrogen receptor-alpha signaling in breast cancer cells. Oncogenesis.

[34] Burandt E., Jens G., Holst F., Janicke F., Muller V., Quaas A., Choschzick M., Wilczak W., Terracciano L., Simon R. (2013). Prognostic relevance of AIB1 (NCoA3) amplification and overexpression in breast cancer. Breast Cancer Res. Treat..

[35] Dubrovska A., Hartung A., Bouchez L.C., Walker J.R., Reddy V.A., Cho C.Y., Schultz P.G. (2012). CXCR4 activation maintains a stem cell population in tamoxifen-resistant breast cancer cells through AhR signalling. Br. J. Cancer.

[36] Shi Y., Riese D.J., Shen J. (2020). The Role of the CXCL12/CXCR4/CXCR7 Chemokine Axis in Cancer. Front. Pharmacol.

[37] Al-Dhfyan A., Alhoshani A., Korashy H.M. (2017). Aryl hydrocarbon receptor/cytochrome P450 1A1 pathway mediates breast cancer stem cells expansion through PTEN inhibition and beta-Catenin and Akt activation. Mol. Cancer.

[38] Gao H., Chakraborty G., Zhang Z., Akalay I., Gadiya M., Gao Y., Sinha S., Hu J., Jiang C., Akram M. (2016). Multi-organ Site Metastatic Reactivation Mediated by Non-canonical Discoidin Domain Receptor 1 Signaling. Cell.

[39] Wang S., Wang N., Zheng Y., Yang B., Liu P., Zhang F., Li M., Song J., Chang X., Wang Z. (2020). Caveolin-1 inhibits breast cancer stem cells via c-Myc-mediated metabolic reprogramming. Cell Death Dis..

[40] Kang Y.P., Yoon J.H., Long N.P., Koo G.B., Noh H.J., Oh S.J., Lee S.B., Kim H.M., Hong J.Y., Lee W.J. (2019). Spheroid-Induced Epithelial-Mesenchymal Transition Provokes Global Alterations of Breast Cancer Lipidome: A Multi-Layered Omics Analysis. Front. Oncol..

[41] Mukherjee A., Kenny H.A., Lengyel E. (2017). Unsaturated Fatty Acids Maintain Cancer Cell Stemness. Cell Stem Cell.

[42] de Sousa E.M.F., Vermeulen L. (2016). Wnt Signaling in Cancer Stem Cell Biology. Cancers.

[43] Lopez-Lazaro M. (2015). The migration ability of stem cells can explain the existence of cancer of unknown primary site. Rethinking metastasis. Oncoscience.

[44] Nallanthighal S., Heiserman J.P., Cheon D.J. (2019). The Role of the Extracellular Matrix in Cancer Stemness. Front. Cell Dev. Biol..

[45] Stelling M.P., Motta J.M., Mashid M., Johnson W.E., Pavao M.S., Farrell N.P. (2019). Metal ions and the extracellular matrix in tumor migration. FEBS J..

[46] Bellomo C., Caja L., Moustakas A. (2016). Transforming growth factor beta as regulator of cancer stemness and metastasis. Br. J. Cancer.

[47] Borgstrom A., Peinelt C., Stoklosa P. (2021). TRPM4 in Cancer—A New Potential Drug Target. Biomolecules.

[48] Rhodes D.R., Yu J., Shanker K., Deshpande N., Varambally R., Ghosh D., Barrette T., Pandey A., Chinnaiyan A.M. (2004). ONCOMINE: A cancer microarray database and integrated data-mining platform. Neoplasia.

[49] Song K., Farzaneh M. (2021). Signaling pathways governing breast cancer stem cells behavior. Stem Cell Res. Ther..

[50] Kotiyal S., Bhattacharya S. (2014). Breast cancer stem cells, EMT and therapeutic targets. Biochem. Biophys. Res. Commun..

[51] Daulat A.M., Bertucci F., Audebert S., Serge A., Finetti P., Josselin E., Castellano R., Birnbaum D., Angers S., Borg J.P. (2016). PRICKLE1 Contributes to Cancer Cell Dissemination through Its Interaction with mTORC2. Dev. Cell.

[52] Kappel S., Stoklosa P., Hauert B., Ross-Kaschitza D., Borgstrom A., Baur R., Galvan J.A., Zlobec I., Peinelt C. (2019). TRPM4 is highly expressed in human colorectal tumor buds and contributes to proliferation, cell cycle, and invasion of colorectal cancer cells. Mol. Oncol..

[53] Loo S.K., Ch’ng E.S., Md Salleh M.S., Banham A.H., Pedersen L.M., Moller M.B., Green T.M., Wong K.K. (2017). TRPM4 expression is associated with activated B cell subtype and poor survival in diffuse large B cell lymphoma. Histopathology.

[54] Sagredo A.I., Sagredo E.A., Pola V., Echeverria C., Andaur R., Michea L., Stutzin A., Simon F., Marcelain K., Armisen R. (2019). TRPM4 channel is involved in regulating epithelial to mesenchymal transition, migration, and invasion of prostate cancer cell lines. J. Cell Physiol..

[55] Rivas J., Diaz N., Silva I., Morales D., Lavanderos B., Alvarez A., Saldias M.P., Pulgar E., Cruz P., Maureira D. (2020). KCTD5, a novel TRPM4-regulatory protein required for cell migration as a new predictor for breast cancer prognosis. FASEB J..

[56] Pattabiraman D.R., Weinberg R.A. (2014). Tackling the cancer stem cells—What challenges do they pose?. Nat. Rev. Drug Discov..

[57] Zardavas D., Irrthum A., Swanton C., Piccart M. (2015). Clinical management of breast cancer heterogeneity. Nat. Rev. Clin. Oncol..

[58] Zheng Q., Zhang M., Zhou F., Zhang L., Meng X. (2020). The Breast Cancer Stem Cells Traits and Drug Resistance. Front. Pharmacol..

[59] Bai X., Ni J., Beretov J., Graham P., Li Y. (2018). Cancer stem cell in breast cancer therapeutic resistance. Cancer Treat. Rev..

[60] Gupta P.B., Onder T.T., Jiang G., Tao K., Kuperwasser C., Weinberg R.A., Lander E.S. (2009). Identification of selective inhibitors of cancer stem cells by high-throughput screening. Cell.

[61] Liu S., Wicha M.S. (2010). Targeting breast cancer stem cells. J. Clin. Oncol..

[62] Yan W., Chen Y., Yao Y., Zhang H., Wang T. (2013). Increased invasion and tumorigenicity capacity of CD44+/CD24- breast cancer MCF7 cells in vitro and in nude mice. Cancer Cell Int..

[63] Biasiotta A., D’Arcangelo D., Passarelli F., Nicodemi E.M., Facchiano A. (2016). Ion channels expression and function are strongly modified in solid tumors and vascular malformations. J. Transl. Med..

[64] Sharpe M.A., Baskin D.S. (2016). Monoamine oxidase B levels are highly expressed in human gliomas and are correlated with the expression of HiF-1alpha and with transcription factors Sp1 and Sp3. Oncotarget.

[65] Lathia J.D., Mack S.C., Mulkearns-Hubert E.E., Valentim C.L., Rich J.N. (2015). Cancer stem cells in glioblastoma. Genes Dev..

[66] Li H., Feng Z., He M.L. (2020). Lipid metabolism alteration contributes to and maintains the properties of cancer stem cells. Theranostics.

[67] Cheng Q., Chen A., Du Q., Liao Q., Shuai Z., Chen C., Yang X., Hu Y., Zhao J., Liu S. (2018). Novel insights into ion channels in cancer stem cells (Review). Int. J. Oncol..

[68] Burris S.K., Wang Q., Bulley S., Neeb Z.P., Jaggar J.H. (2015). 9-Phenanthrol inhibits recombinant and arterial myocyte TMEM16A channels. Br. J. Pharmacol..

[69] Scheel C., Weinberg R.A. (2012). Cancer stem cells and epithelial-mesenchymal transition: Concepts and molecular links. Semin. Cancer Biol..

[70] VanderVorst K., Dreyer C.A., Konopelski S.E., Lee H., Ho H.H., Carraway K.L. (2019). Wnt/PCP Signaling Contribution to Carcinoma Collective Cell Migration and Metastasis. Cancer Res..

[71] Arullampalam P., Preti B., Ross-Kaschitza D., Lochner M., Rougier J.S., Abriel H. (2021). Species-Specific Effects of Cation Channel TRPM4 Small-Molecule Inhibitors. Front. Pharmacol..

[72] Low S.W., Gao Y., Wei S., Chen B., Nilius B., Liao P. (2021). Development and characterization of a monoclonal antibody blocking human TRPM4 channel. Sci. Rep..

