# Supplementary material for: Transcriptional Profiling of Tumorspheres Reveals TRPM4 as a Novel Stemness Regulator in Breast Cancer
Source: Biomedicines. 2021 Oct 1;9(10):1368. doi: 10.3390/biomedicines9101368 (PMC8533210; doi:10.3390/biomedicines9101368)
Supplement: Supplementary file 1 [file biomedicines-09-01368-s001.zip › Supplementary data/Supplementary material.pdf]

Supplementary Materials

Transcriptional profiling of tumorspheres reveals TRPM4 as a novel stemness regulator in breast cancer

John Verigos[1,2], Dimitris Kordias[1,2#], Styliani Papadaki[2#] and Angeliki Magklara [1,2,3*]

Figure S1. Supplementary Figure 1. MCF-7 mammospheres are highly enriched in b-CSCs. (A) FACS analysis of MCF-7 cells grown in 2D monolayers or in 3D mammospheres using antibodies against CD44 and CD24. (B) Quantification of the FACS results. (C) mRNA expression levels of known stemness markers in mammospheres. The fold-change of mRNA expression over the adherent cells is shown.

Figure S2. Expressed genes in MCF-7 cells grown as adherent monolayers or mammospheres. Out of the 27,967 genes that were analysed, 14,641 and 14,937 were expressed in the adherent monolayer cells and mammospheres, respectively. No expression depicts genes that have less than 10 reads in either phenotype.

Figure S3. Gene ontology analysis for DEGs. The top overrepresented biological pathways based on the number of genes for the (A) upregulated and (B) downregulated genes in MCF-7mammospheres resulting from PANTHER analysis.

Figure S4. Oncomine analyses of breast cancer databases. Box plots show that TRPM4 is overexpressed in aggressive breast carcinomas: (A) Curtis breast cancer database G. Invasive Lobular breast Carcinoma vs. Normal, Fold change = 1.602, Tubular Breast Carcinoma vs. Normal, Fold change = 1.524, Mucinous Breast Carcinoma vs. Normal, Fold change = 1.468, Invasive Ductal and Invasive Lobular Breast Carcinoma vs. Normal, Fold change = 1.406 and Invasive Ductal Breast Carcinoma vs. Normal, Fold change = 1.439 (p-value<0.001). (B) Ma breast cancer database. Ductal Breast Carcinoma vs. Normal, Fold change = 1.413 and Invasive Ductal Breast Carcinoma vs. Normal, Fold change = 1.202 (p-value≤0.005). Numbers in parentheses indicate the number of samples. The y-axis represents log2 median-centered intensity (normalized expression). Shaded boxes represent the interquartile range (25th–75th percentile). Whiskers represent the 10th–90th percentile. The bars denote the median (adapted from www.oncomine.org).

Figure S5. Western blot analysis for TRPM4 siRNA knock-down in MCF-7 cells.

Legends for the supplementary tables

Table S1. The top 200 genes expressed in MCF-7 adherent monolayer cells and mammospheres. Gene ranking was based on the number of reads and the top 100 genes for each group were selected. Genes in colour and in bold are discussed in the text.

Table S2. The 1,421 differentially expressed genes (DEGs) between MCF-7 adherent monolayer cells and mammospheres identified by RNA-sequencing. The transcriptomes of adherent monolayer cells and mammospheres were compared using the R package DESEQ2 and DEGs were identified by setting a 2-fold-change and a p-value≤0.05 (with p-adj≤0.01) as cut-off values.

Table S3. The genes in each biological pathway overrepresented in DEGs. PANTHER GO-Slim biological process analysis was performed and the genes from the most overrepresented pathways are presented.

Table S4. The genes of the enriched hallmarks in DEGs. Hallmark analysis was performed using the GSEA software.

Figure S1.

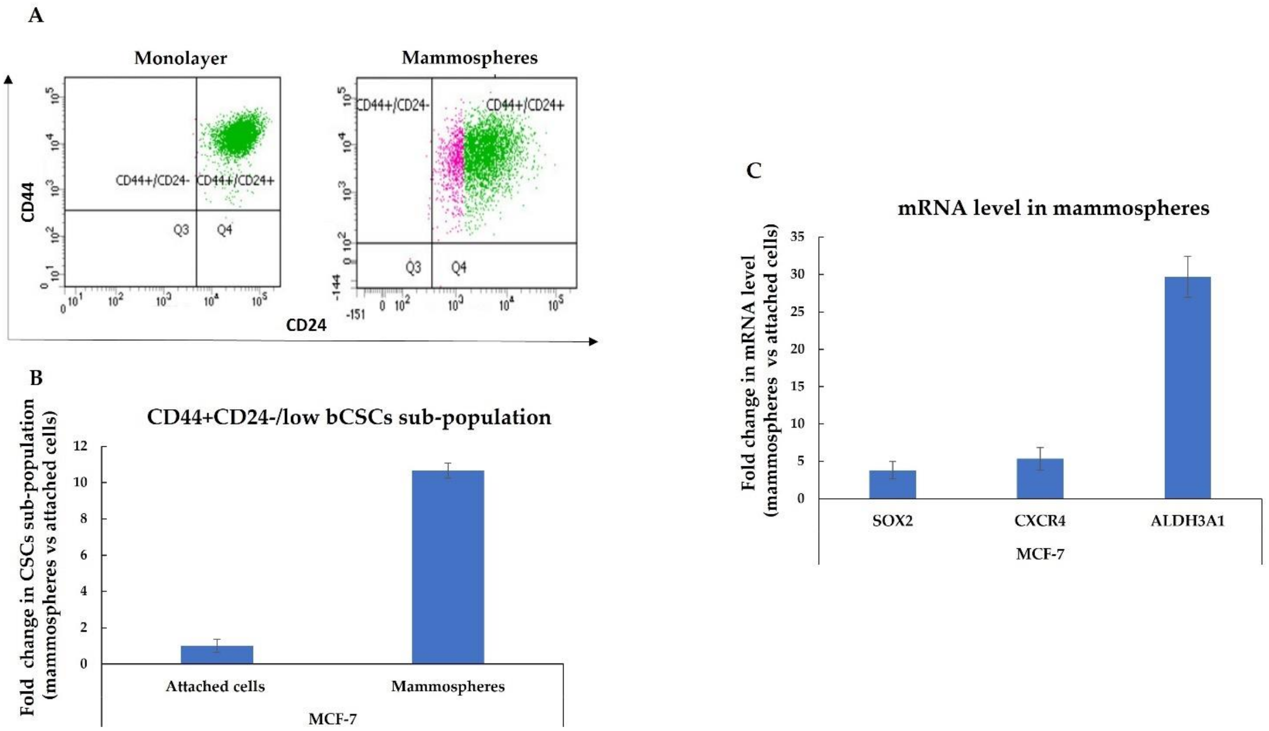
A
Monolayer
Mammospheres
CD44+/CD24-
CD44+/CD24+
Q3
Q4
CD44
CD24
B
CD44+CD24-/low bCSCs sub-population
Fold change in CSCs sub-population (mammospheres vs attached cells)
Attached cells
Mammospheres
MCF-7
C
mRNA level in mammospheres
Fold change in mRNA level (mammospheres vs attached cells)
SOX2
CXCR4
ALDH3A1
MCF-7

Figure S2.

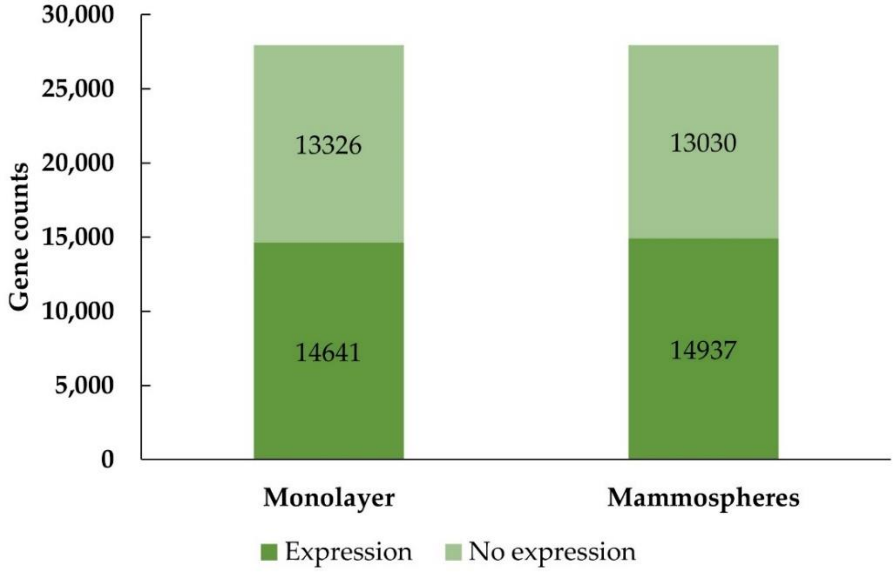
30,000
25,000
20,000
15,000
10,000
5,000
0
Gene counts
13326
14641
13030
14937
Monolayer
Mammospheres
Expression
No expression

Figure S3.

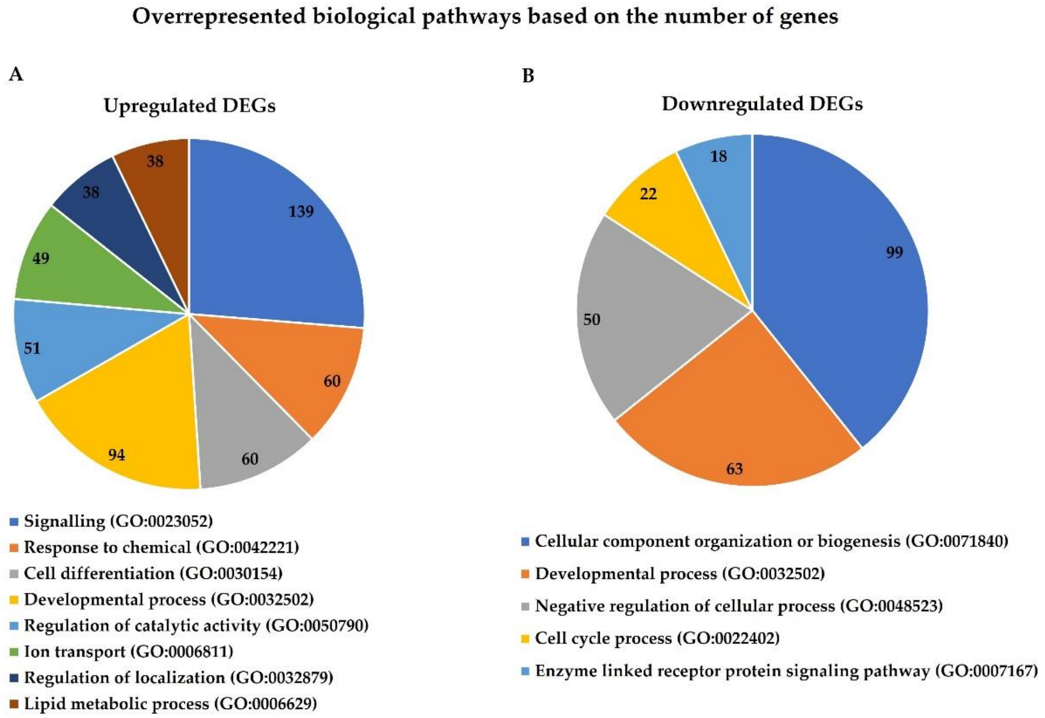
Overrepresented biological pathways based on the number of genes
A
Upregulated DEGs
139
60
60
94
51
49
38
38
Signalling (GO:0023052)
Response to chemical (GO:0042221)
Cell differentiation (GO:0030154)
Developmental process (GO:0032502)
Regulation of catalytic activity (GO:0050790)
Ion transport (GO:0006811)
Regulation of localization (GO:0032879)
Lipid metabolic process (GO:0006629)
B
Downregulated DEGs
99
63
50
22
18
Cellular component organization or biogenesis (GO:0071840)
Developmental process (GO:0032502)
Negative regulation of cellular process (GO:0048523)
Cell cycle process (GO:0022402)
Enzyme linked receptor protein signaling pathway (GO:0007167)

Figure S4.

A

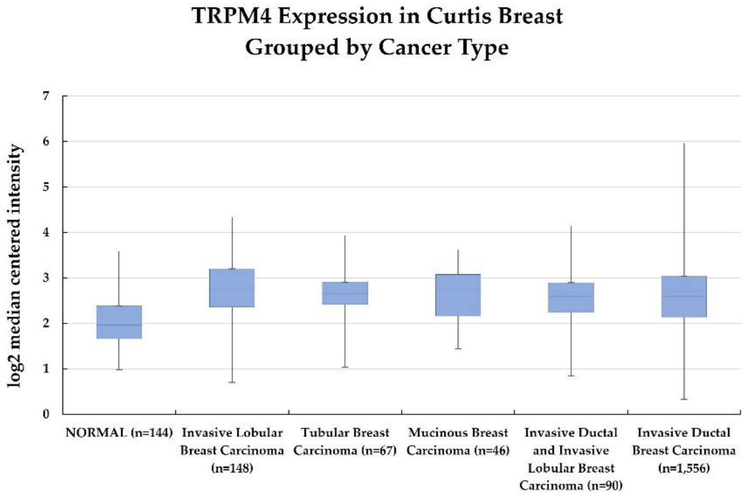
TRPM4 Expression in Curtis Breast
Grouped by Cancer Type
log2 median centered intensity
7
6
5
4
3
2
1
0
NORMAL (n=144)
Invasive Lobular Breast Carcinoma (n=148)
Tubular Breast Carcinoma (n=67)
Mucinous Breast Carcinoma (n=46)
Invasive Ductal and Invasive Lobular Breast Carcinoma (n=90)
Invasive Ductal Breast Carcinoma (n=1,556)

B

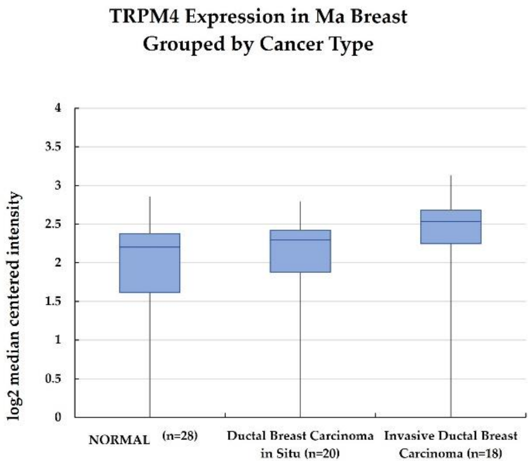
TRPM4 Expression in Ma Breast
Grouped by Cancer Type
log2 median centered intensity
4
3.5
3
2.5
2
1.5
1
0.5
0
NORMAL (n=28)
Ductal Breast Carcinoma in Situ (n=20)
Invasive Ductal Breast Carcinoma (n=18)

Figure S5.

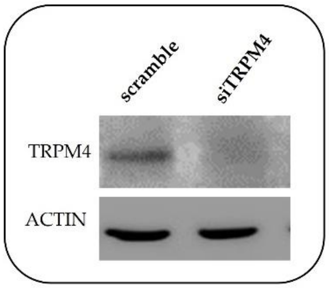

Tables

Table S1.

**Top 100 expressed genes in MCF-7 monolayers**	***KRT8***, *EEF1A1*, *GAPDH*, ***EEF2***, *CSDE1*, *ACTB*, ***KRT18***, *PABPC1*, *PKM*, *RNR2*, *GNAS*, *ACTG1*, *HSP90AA1*, *CTSD*, *RPS2*, *RPL8*, *KRT19*, *RPL4*, *TPT1*, *RPS3*, *FTH1*, *ALDOA*, *CLTC*, *RPLP0*, *EEF1G*, *RPL13*, *PTMA*, *HNRNPA2B1*, *HSP90AB1*, *NCOA3*, *SULF2*, *TRIM37*, *RPL7A*, *HSPA8*, *RACK1*, *HSPB1*, *TUBB*, *SCD*, *PSMD6*, *SPTSSB*, *NPM1*, *NUCKS1*, *RPL12*, *EEF1A2*, *ENO1*, *RPS4X*, *H3F3B*, *TUBA1B*, *CFL1*, *FLNA*, *YWHAZ*, ***GATA3***, *NCL*, *RPL3*, *DDX5*, *RPL32*, *PPIA*, *RPS6KB1*, *TMBIM6*, *RPL30*, *TPM4*, *BCAS3*, *MYL6*, *RPS11*, *RPS18*, *EIF4G1*, *EIF4G2*, *TMSB4X*, *RPL15*, *RPS24*, *RPS6*, *RPL11*, *P4HB*, *RPL6*, *CCND1*, *HNRNPK*, *RPLP1*, *CANX*, *CDH1*, *RPS25*, *RPL41*, *XBP1*, *PRSS23*, ***FASN***, *PCBP2*, *RNR1*, *EIF4B*, *APPBP2*, *YBX1*, *RPS8*, *SLC9A3R1*, *PFN1*, *HNRNPC*, *SET*, *PSAP*, *RPL7*, *SLC39A6*, *RPL10*, *HDLBP*, *TUBB4B*
**Top 100 expressed genes in MCF-7 mammospheres**	***KRT8***, *RNR2*, *GNAS*, *PKM*, *SCD*, *EEF1A1*, *SULF2*, *GAPDH*, ***KRT18***, *CSDE1*, *KRT19*, *ENO1*, ***EEF2***, *ACTB*, *HSP90AA1*, *SLC39A6*, *ACTG1*, *NCOA3*, *ALDOA*, *CLTC*, *TPT1*, *PABPC1*, *FTH1,P4HB*, *DDX5*, *RNR1*, ***FASN***, *TMBIM6*, *PTMA*, *PSMD6*, *HNRNPA2B1*, *ATP1A1*, *CTSD*, *RPL8*, *S100A11*, *SPTSSB*, *YWHAZ*, ***GATA3***, *HSP90AB1*, *EEF1A2*, *CLU*, *EEF1G*, *BMP7*, *RPL4*, *XBP1*, *SPINT2*, *TRIM37*, *PSAP*, *RPL13*, *RPS2*, *HSPA8*, *NUCKS1*, *CFL1*, *PPIA*, *RPL7A*, *RPLP0*, *EIF4G1*, *APP*, *RACK1*, *MYL6*, *CANX*, *RPS3*, *PLEC*, *H3F3B*, *CDH1*, *FLNA*, *SYTL2*, *CYP1B1*, *SRRM2*, *HSPB1*, *RPS4X*, *DHCR24*, *CD63*, *LY6E*, *PRKAR1A*, *EIF4G2*, *NPM1*, *HDLBP*, *APPBP2*, *TUBB*, *TMSB4X*, *HNRNPK*, *NCL*, *PHLDA1*, *TBC1D9*, *CALR*, *HSPA5*, *COX6C*, *PCBP2*, *RPS6KB1*, *UBB*, *CALM1*, *RPL15*, *TUBA1B*, *PLK2*, *ZNF217*, *BSG*, *CRABP2*, *UBC*, *RPS11*

Table S2. See the excel file entitled **“Suppl. Table S2”**

Table S3. See the excel file entitled **“Suppl. Table S3”**

Table S4. See the excel file entitled **“Suppl. Table S4”**
